# Detection and characterization of small-sized microplastics (≥ 5 µm) in milk products

**DOI:** 10.1038/s41598-021-03458-7

**Published:** 2021-12-15

**Authors:** Paulo A. Da Costa Filho, Daniel Andrey, Bjorn Eriksen, Rafael P. Peixoto, Benoit M. Carreres, Mark E. Ambühl, Josep B. Descarrega, Stephane Dubascoux, Pascal Zbinden, Alexandre Panchaud, Eric Poitevin

**Affiliations:** Société des Produits Nestlé S.A. Nestlé Research, Route du Jorat 57, Lausanne, Switzerland

**Keywords:** Environmental sciences, Chemistry, Optics and photonics

## Abstract

Microplastics (MPs) have gained a high degree of public interest since they are associated with the global release of plastics into the environment. Various studies have confirmed the presence of MPs throughout the food chain. However, information on the ingestion of MPs via the consumption of many commonly consumed foods like dairy products are scarce due to the lack of studies investigating the “contamination” of this food group by MPs. This lack of occurrence data is mainly due to the absence of robust analytical methods capable of reliably quantifying MPs with size < 20 µm in foods. In this work, a new methodology was developed to accurately determine and characterize MPs in milk-based products using micro-Raman (μRaman) technology, entailing combined enzymatic and chemical digestion steps. This is the first time that the presence of relatively low amounts of small-sized MP (≥ 5 µm) have been reported in raw milk collected at farm just after the milking machine and in some processed commercial liquid and powdered cow’s milk products.

## Introduction

MPs are generally defined as plastic debris particles less than 5 mm in size^1,2^. Despite the lack of an internationally agreed definition of the lower size limit for MPs, a minimum size of 1 µm is in accordance with the SI nomenclature for MPs category^3^.

Large amounts of MPs are known to accumulate in the environment mainly due to them being directly discharged from land-based sources directly into the marine environment^4^, including oceans^5,6^, freshwaters^7^ and finally waste waters^8^. Concerns about MP pollution of terrestrial ecosystems are also increasing after proof of their presence in soils^9,10^.

The detection of MPs in environment and related biota is however challenging. In the past 10 years, the use of more performant Fourier transform infra-red^11–13^ or Raman micro-spectroscopy^14,15^ compared to pyrolysis–gas chromatography-mass spectrometry (Py-GC–MS)^16,17^ and thermal desorption-gas chromatography–mass spectrometry (TD-GC–MS)^18^ have shown the presence of MPs, mainly in the size range from 20 to 250 µm in aquatic fauna, including some common species consumed by humans^19–24^. Apart from marine species, recent scientific review papers have highlighted the unequivocal presence of MPs in a wider variety of foods intended for human consumption^25^. These foods include poultry meat, snails, edible salts, sugar, honey, and various beverages like drinking and bottled waters, soft drinks, cold tea, beer and liquid milk products^26–36^. Successful identification of small-sized MPs (ca 5 µm) in drinking waters^37,38^ and recent reviews on the occurrence of MPs in a wide range of foodstuffs have led to increased media interest and concerns about this emerging issue among some international agencies and public health organizations^39–43^.

The analytical methods published to date on the analysis of MPs in food show several shortcomings. Many of the methods are either not adequately sensitive to identify MPs with size < 20 µm and/or specific enough to discriminate organic particles from MPs. They are also simply incomplete in terms of performance by lack of information on the MPs size range and on polymer identification^44–46^.

These difficulties can be explained by the numerous analytical challenges in isolating intact MPs after destruction of the organic food matrix^47,48^. According to some papers on seafood sample preparation for MPs analysis, the method of choice for purification of a sample from organic matter that proved to be cost-and time-effective, is an alkaline digestion approach using dilute potassium hydroxide solution for 48–72 h with a relative limited degradation of main polymers^49,50^. Enzymatic methodology also proved to be a promising alternative in degrading the organic matter, especially tissues while not affecting the polymers’ integrity^51^. Currently, μRaman amongst all other techniques is considered as the most sensitive technique for MPs analysis after sample digestion, allowing the detection of particles of less than 1 ng in weight and 1 µm in size thanks to the high spatial resolution of the laser beam^52–55^.

The scarcity of data in food commodities in general prompted multinational corporations from food and beverages industry to study the occurrence of MPs in selected food materials (e.g. raw milk, salt, sugar, honey, etc.). Raw milk is considered as main natural source of many of the valuable nutrients (proteins, lipids, vitamins and minerals). Therefore, fresh milk is still one of the most consumed raw material worldwide, corresponding to a global production reaching nearly 906 million tons in 2020 with 8.7% of the production being traded by dairy industry as milk products^56^. The level of contamination of MPs in milk products remains largely unknown due to the scarcity of published scientific data, and associated difficulty to isolate and quantify MPs in such complex food matrix^35,36^. Along a dairy supply chain, contamination with MPs can occur at various stages. They can be introduced during the milking of the cow at the farm, during downstream processing, or via the final packaging^57^.

In this study, a new methodology was developed and validated for 5 different polymers (polypropylene (PP), polyethylene (PE), polystyrene (PS), polyamide (PA) and polymethylmethacrylate (PMMA)) constituting small-sized MPs. This methodology was applied in samples of cow's milk using a fast and efficient MPs extraction. This method is based on a two-step preparation that involves multi-enzymatic digestion followed by hot alkaline hydrolysis and a final filtration step through a silicon (Si) filter.

The material retained on the filter was subsequently analyzed using high resolution μRaman equipment.

The collected spectra were then processed using a custom-made software to reliably characterize for the first time micro-sized MPs (≥ 5 µm) in farm’s milk and processed cow’s milk products using high resolution μRaman and SEM–EDX technologies.

## Results

### Mitigating laboratory contamination

**Figure 1:**
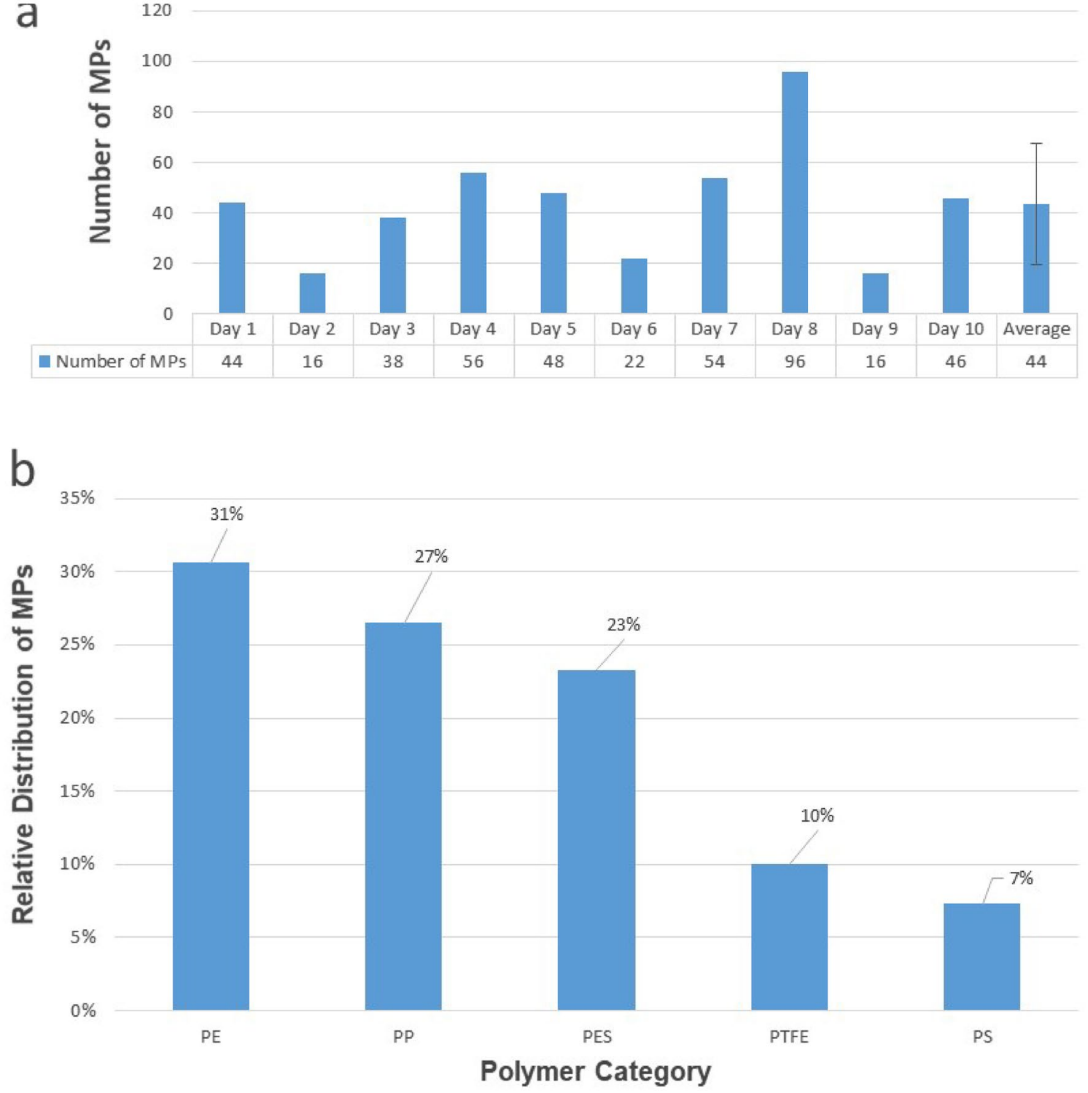
Monitoring of MPs contamination in method blanks. (**a**) Number of MPs in 10 procedural blanks; (**b**) relative distribution of MPs in the method blanks based on pol3: ymer type.

Method blanks performed during method development revealed different sources of MPs contamination. These were mainly coming from the laboratory environment, chemical reagents, containers and laboratory clothing. These sources of contamination were subsequently minimized following the mitigating actions described in the “Materials and Methods” section. The total number of MPs found in a set of 10 different daily method blanks used for this study (Fig. 1a, b) ranged from 16 to 96 MPs retained on the filter, with an average of 44 ± 24. PE was the most frequently found polymer in method blanks representing ca. 31% of the total amount followed by PP (27%), polyester (PES) (23%) and in smaller amounts of polytetrafluoroethylene (PTFE) and PS. PA and polyurethane (PU) polymers represent less than 2% of total MPs identified. More than ca 80% of MPs found in the set of 10 blanks had a measured surface area of less than 50 µm^2^ (ca particle size ≤ 7 µm).

### Method recovery and polymer integrity after food matrix digestion

Samples of ultrapure water and two pasteurized liquid cow’s milk samples (brand B, whole milk 2, 3.5% fat and brand C, skimmed milk, 0.1% fat) were spiked with commercial standards of five different MPs to determine the recovery rate of the method and to check the potential degradation of MP standards after hot alkaline digestion.

Table 1 shows the recovery of the 5 standards of MPs spiked in ultrapure water sample without digestion before filtration and in a cow’s milk sample (brand B, whole milk, 3.5% fat) following the whole method procedure (i.e. digestion before filtration and Raman analysis). Particle recovery for all 5 different polymer standards (PMMA and PS beads, PE, PP and PA polymers with size ranging from 5 to 40 µm) ranged from 78 to 141% with relative standard deviation ranging from 12 to 45%. These results were acceptable given the challenging procedure to prepare repeatable standard polymer solutions with low concentrations of MPs.

**Table 1:** Recovery rates of 5 MP standards in ultra-pure water and liquid cow milk (brand B, whole milk 2, 3.5% fat).

Spiked samples^a^	PMMA (%)	PS (%)	PP (%)	PE (%)	PA (%)
Ultra-pure water (N = 7) without digestion	78 ± 12	97 ± 13	90 ± 18	86 ± 19	94 ± 24
Liquid cow’s whole milk 2 (brand B, 3.5% fat) (N = 4) with digestion	97 ± 17	123 ± 45	136 ± 34	119 ± 30	141 ± 12

^a^After blank subtraction of PP, PE and PS contaminations.

N represents the number of replicates.

**Figure 2:**
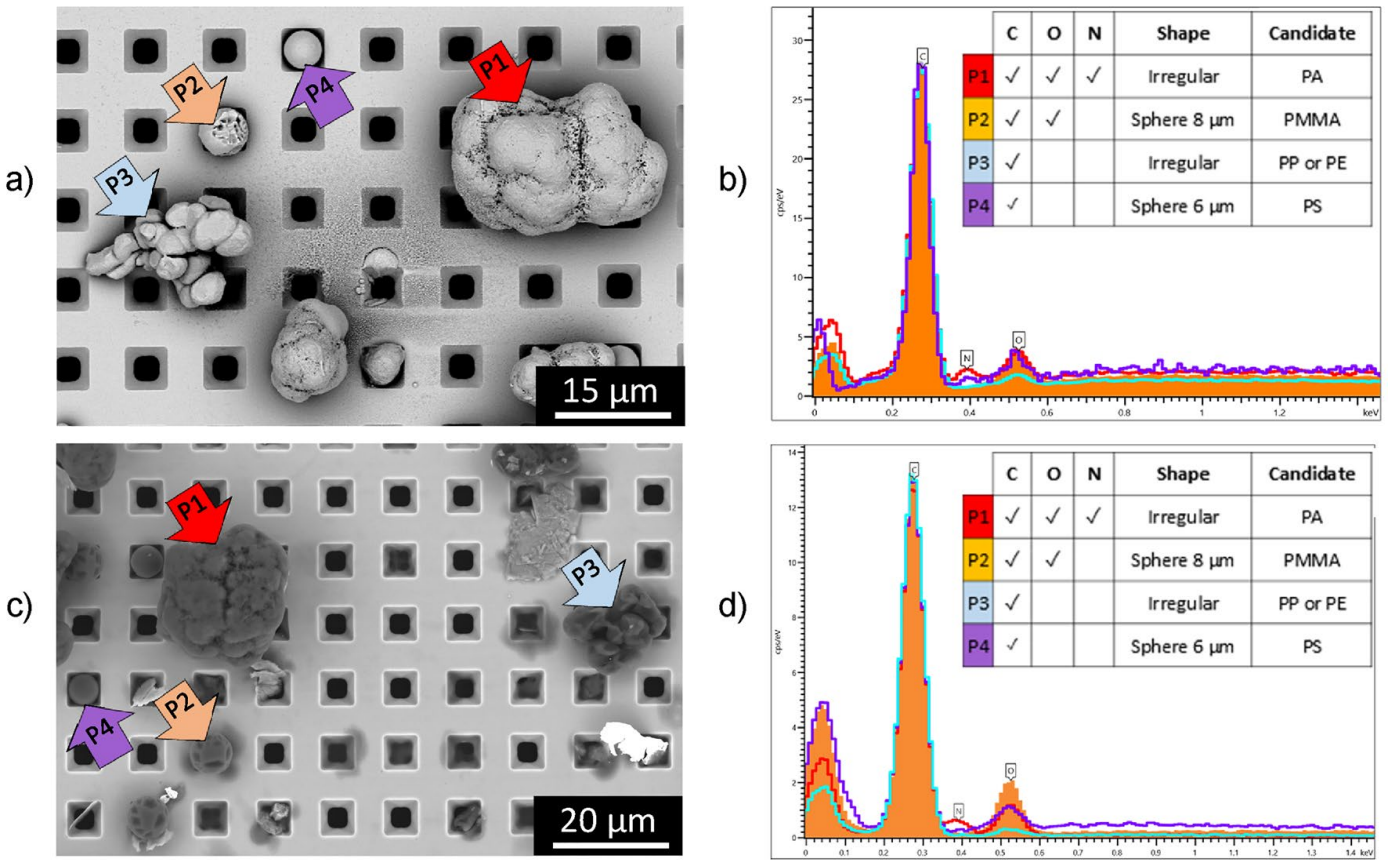
SEM–EDX analysis of MPs in ultrapure water: (**a**) picture showing a SEM micrograph of a spiked water sample with PA polymer (red arrow P1), PMMA polymer (orange arrow P2), olefins polymers PE–PP (blue arrow P3) and PS polymer (purple arrow P4); (**b**) EDX spectra of the MP standards pointed out in panel 2a; (**c**) SEM micrograph of the milk sample (brand C, skimmed milk, 0.1% fat) spiked with the same polymers; (**d**) EDX spectra of the MP standards selected in panel 2c.

Figure 2 depicts SEM images of a spiked ultrapure water sample (Fig. 2a MP standards, no digestion before filtration involved) and of a spiked milk sample (brand C, skimmed milk, 0.1% fat) (Fig. 2c, after digestion), as well as EDX spectra of encountered particles (Fig. 2b, d representing the water solution and cow’s milk sample respectively).

The polymers PA, PMMA, PS and polyolefins (PE-PP) were identified in the spiked water sample (Fig. 2a, b) by combining the morphology found in the SEM micrographs and the identification of the major chemical elements via EDX detection. These 4 groups of MP standards (PA, PMMA, PP-PE and PS) were also detected in spiked milk after digestion (Fig. 2c, d) since their morphology was well preserved after digestion despite their small sizes.

**Figure 3:**
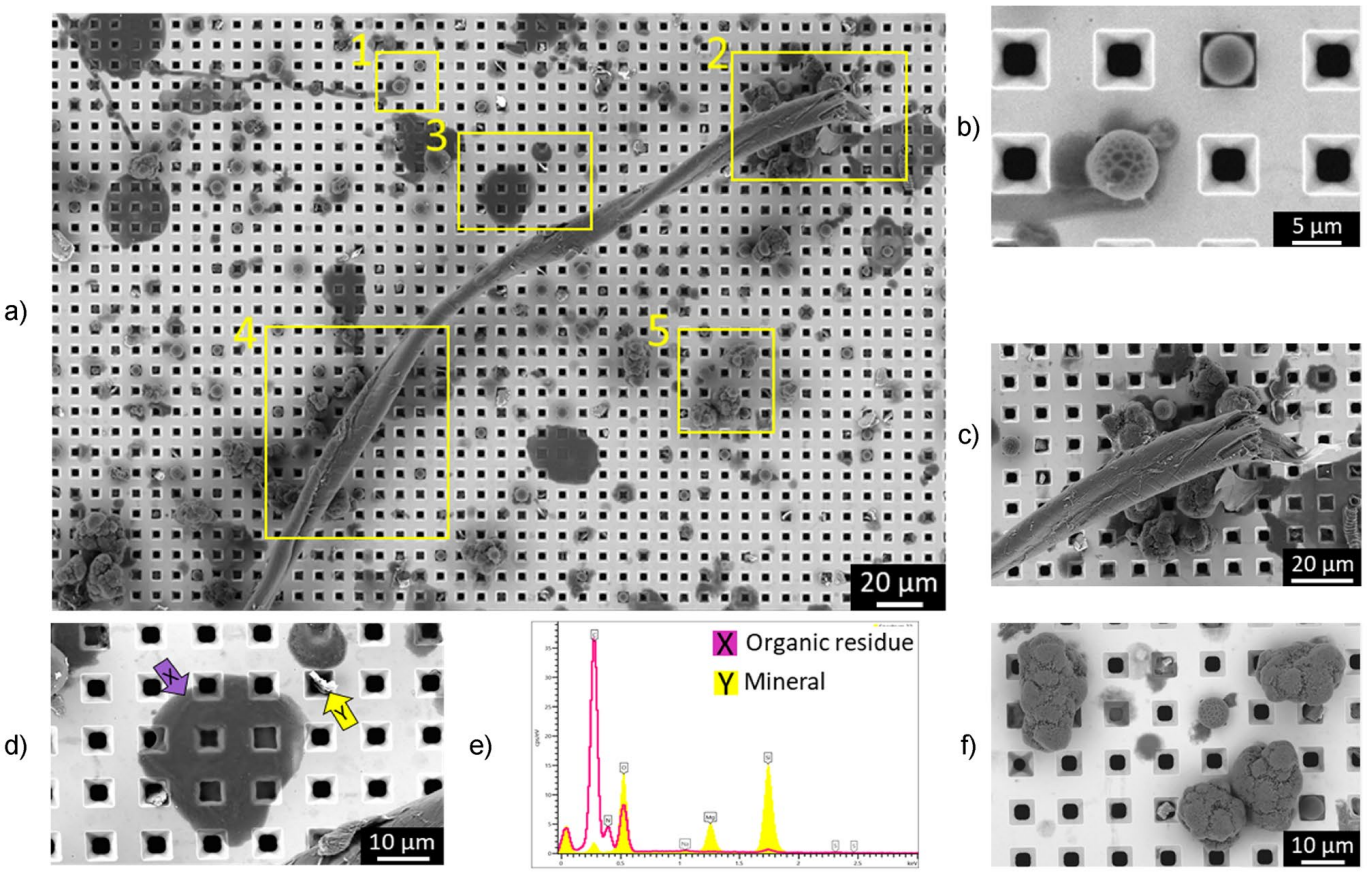
SEM–EDX analysis of a part of the filtered area for a spiked commercial cow’s milk sample (brand C, skimmed milk, 0.1% fat) showing: (**a**) different yellow zones (numbered 1 to 5) with different types of particles identified in each zone. Zone 1 = PS and PMMA beads; zone 2 = undigested organic fiber with agglomerates of other particles including polymer standards; zone 3 = organic residue and mineral particle; zone 4 = undigested fiber; zone 5 = polymer beads (PMMA, PS) and PA particles; (**b**) magnified zone 1 showing PS and PMMA beads; (**c**) magnified zone 2 showing agglomerates of PMMA, PS and PA particles stuck to end of the fiber; (**d**) magnified zone 3 showing some organic residue and mineral particles; (**e**) EDX spectrum on the organic residue (X) and mineral particle (Y) present in panels D; (**f**) magnified zone 5 showing PA particles and PMMA/PS beads.

SEM imaging of the Si filter surface of a spiked milk sample (brand C, skimmed milk, 0.1% fat) provided an overview of the particle structures present after alkaline digestion (Fig. 3). The high-resolution SEM images of the digested sample showed the filter surface at a submicron level and qualitatively distinguishes between the different types of particles (fibers, organic residues, mineral particles and standards of MPs) present after sample digestion. No significant differences were observed in the MP standards after digestion, neither in morphology (via SEM) nor in chemical composition (via EDX analysis).

For example, in the SEM images of a Si Filter surface, 4 particle shapes were identified (Fig. 3a). These were assigned to undigested fibrous fragments, digested organic residue, mineral particles, and to the retained MP standards (PA particles, PS and PMMA beads). The MP standards were widely distributed over the entire surface of the filter, even when some MPs were agglomerated with some fibers (Fig. 3c).

Mineral based particles (e.g. Mg in Fig. 3d) can be identified through EDX analysis, but fibers, organic residues and MPs show similar EDX spectra for C, O and N elements (Fig. 3e). Therefore, the identification of MPs using only EDX information is very challenging and could only be applied to PMMA and PS particles added intentionally with well-defined morphology (e.g. beads with single particle size, Fig. 3b, f).

### Validation of the Raman methodology

**Figure 4:**
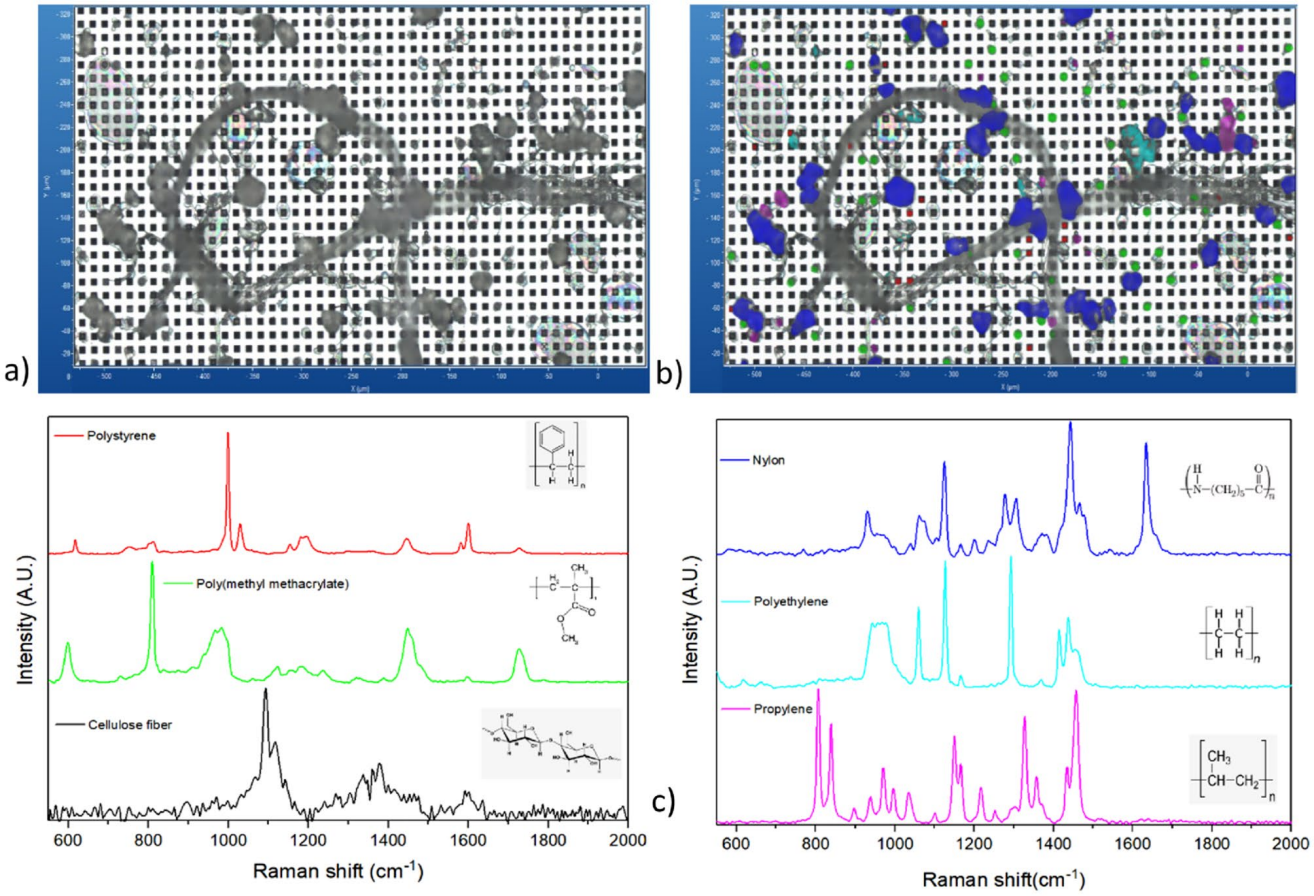
µRaman analysis of a part of the filtered area for a cow’s milk (brand C, skimmed milk, 0.1% fat) spiked with 5 standards of MPs: (**a**) optical microscope image of digested particles on a part of the Si filter; (**b**) Same image with MPs and cellulose fiber detected and identified (PS colored in red, PMMA in green, cellulose fiber in gray, PA in blue, PE in cyan and PP in pink); (**c**) related colored Raman spectra collected from 5 MP standards and cellulose fiber after sample digestion.

Figure 4 depicts the optical microscope image of surface part of a Si filter for a spiked pasteurized commercial cow’s milk sample (brand C, skimmed milk, 0.1% fat) obtained after alkaline digestion and the Raman spectrum of each type of MP standard detected. This image was obtained using an optical microscope equipped with bright-field illumination (Fig. 4a). The same image was processed using the software of LabSpec 6.5 to highlight the cellulose fiber and the 5 different types of MPs present in the sample (Fig. 4b). In addition, the Raman spectrum of each MP present is shown in Fig. 4c. The Raman spectra (Fig. 4c) were easily attributed to each polymer using the specific vibrational assignment of the main peaks (Table S1) with a good fit when compared to a reference database (KnowItAll version 18.3.111.0 Raman spectral library). Importantly, no significant physico-chemical changes were also identified by µRaman in the polymer structures after alkaline digestion.

### Microplastics determination in cow’s milk samples

The Raman method was successfully applied to detect and quantify the number of MPs in liquid and reconstituted cow’s milk samples. The concentration of MPs identified in milk samples ranged from 204 to 1004 MPs per 100 mL of sample. The surface area of these MPs was mainly ≤ 50 µm^2^ representing between 69 and 89% of the total detected MPs in milk samples (Table 2).

**Table 2:** Number and type of MPs detected in cow’s milk samples.

Milk samples	Total number of MPs per 100 mL**	MP surface < 50 µm^2^ (%)	PE	PES	PP	PTFE	PS	Other polymers
Blank (milking machine)—(2nd collection)	52	81	52	ND	ND	ND	ND	ND
Raw milk (1st collection)	204	70	76	44	76	ND	ND	4 PU*** 4 PA***
Raw milk (2nd collection)	625	71	585	10	0	25	ND	5 PA***
Cow’s milk liquid (brand A)	232	83	ND	8	20	108	96	ND
Cow’s milk liquid 1 (brand B)	284	85	52	44	20	116	28	4 PU*** 4 PSU*** 16 PVA***
Cow’s milk liquid 2 (brand B)	348	69	136	100	30	24	38	20 PU***
Cow’s milk liquid (brand C)	172	89	44	ND	32	84	ND	12 PU***
Cow’s milk powder* (brand A)	356	79	28	188	84	52	ND	4 PA***
Cow milk powder* (brand B)	1004	82	44	32	44	880	ND	4 PA***

*ND* not detected.

*Reconstituted cow’s milk samples (13 g powder in 100 mL water which corresponds to the serving size of liquid and reconstituted cow’s milk samples with a standard nutritional energy of ca 67 kcal for milk products).

**Values subtracted from the blank.

***PA: polyamide; PU: polyurethane; PSU: polysulfone; PVA: polyvinyl alcohol.

The main MPs found in farm milk, processed liquid milk and reconstituted powdered milk samples were PE, PES, PP, PTFE and PS particles and in smaller amounts PA, PU, PSU and PVA polymers, as depicted in Table 2. The two liquid samples 1 and 2 (brand B) contained additionally PU particles. One liquid sample also contained small amounts of PSU and PVA polymers that were not included in the custom-made software but identified by KnowItAll software. The same order of MPs concentration was found in both farm milk and liquid cow’s milk samples. Milk powders (brands A and B) after water reconstitution contained relatively higher levels of MPs compared to farm milk and ready-to-drink milk samples (brands A, B and C). Based on these limited data, it was observed that the number of MPs tended to increase from farm’s milk to processed milk powders, even if the MPs concentrations remained in the same order of magnitude.

## Discussion

The validated digestion procedure used in this study entailed a rapid combined enzymatic and hot alkaline digestion, that effectively removed milk components, hydrolyzing proteins (e.g. whey proteins and caseins), lipids (e.g. triglycerides) and carbohydrates (e.g. lactose) and other organic complex aggregates without degrading the 5 types of MPs that were tested. The step of chemical soft alkaline microwave hydrolysis based on effective chemical solubilization of the milk sample by a quaternary ammonium salt allows a final rapid filtration in cutting all organic matrix in micrometric fragments without significant sample dilution^58^. Another advantage to consider for this rapid digestion procedure, is the size of the resulting digested fragments. With the digested fragments being as small as possible, this increases the efficiency of the microfiltration of the digested milk sample, which would have otherwise lead to clogging of the 5 μm-porosity Si filter^35,36^. In a recent study, it was shown that during the first few minutes of lipase digestion of milk, small fragments of triglycerides (ranging from 1 to 10 μm) are released and subsequently re-aggregate to a higher size (> 50 μm) if digestion time was prolonged^59^. The formation of these so-called “calcium containing soaps” particles is significantly reduced in this digestion procedure by addition of a calcium chelator, thereby avoiding the rapid clogging of the filter.

Method recovery was acceptable for the spiked cow’s milk and water samples. In general, this study showed that the recovery of MPs in replicates of samples of cow's milk after the digestion procedure is generally over 100%. In contrast, the recovery of MPs in undigested water replicates was less than 100%.

The lower recovery obtained for spiked water sample could be linked—due to their hydrophobicity- to the formation of MPs aggregates larger in size than that of added standard MPs particles (Fig. 2a—blue arrow P3). Even if MPs in undigested spiked water were evenly distributed on the Si filter, these aggregates identified as being larger particles than the polymer standard resulted in the total number of particles being finally underestimated after extrapolation of the counting for the total surface.

Higher recovery results found for digested spiked milk samples can eventually be explained by a combination of systematic error during the spiking procedure (e.g. homogenization of standard solution, pipetting, etc.) and an overestimated extrapolation factor to determine the number of microplastics throughout the filter. In fact, many MPs particles were stuck to undigested fibers like cellulose, generating “hot spot” regions. The physical phenomenon that leads MPs to aggregate to fibers was not studied in this work. However, this phenomenon was likely to be associated with strong electrostatic interactions among the MPs and these organic particles. Since the total number was determined in the central half area analyzed where larger organic fragments are present, MPs particles had an uneven distribution and were consequently slightly overestimated due to presence of these aggregates. Therefore, an experiment using 4 replicates of liquid cow’s milk spiked with MPs was conducted to assess whether the extrapolation factor applied to estimate the number of MPs retained in 100% of the filter could lead to their overestimation or underestimation. Figure S3 shows one replicate of cow’s milk spiked with 5 types of MPs (PE, PA, PP, PS and PMMA). The detected MPs and cellulose fibers are highlighted with different colors, as indicated in the legend.

The mapping image of each replicate was divided into 4 equal sub-areas (A1, A2, A3 and A4) of 4 mm^2^ as shown in Fig. S3. The number of particles of each type of microplastic found in A1, A2, A3 and A4, as well as in the entire filtered area, is shown in Table S4.

Table S5 shows the ratio between the number of particles of a given polymer in 100% and 50% of filtered area. All different combination of 2 sub-areas from a set of 4 sub-areas were used to calculate the 50% of filtered area. Since the ratio between the polymers found in 100% and 50% of analyzed filtered area is approximately 2 for the 4 analytical replicates (see Table S5), it can be concluded that there is a similar distribution of particles in 100% and 50% of the filter. Therefore, these results suggest that the risk of overestimation or underestimation can be neglected and that extrapolation factor of 2 can be used for analysis.

In addition, the recovery experiment showed that point-to-point Raman mapping is very efficient for detecting MPs isolated or bound to large particles or microfibers (Fig. 4a). This method can also be optimized to detect particles up to 1 µm in size. However, the main limitation of this approach will be the analysis time required for large surfaces. For example, it takes 20 h to analyze 50% of the surface (i.e. 7 mm^2^) using a spatial resolution of 5 µm. The analysis time must be multiplied by a factor of 25 if the spatial resolution is changed from 5 to 1 µm. Consequently, this approach is not suitable for large surfaces when the spatial resolution is greater than 5 µm.

The leading suppliers of µRaman offer recognition software packages that allow locating and recognizing particles. This approach is interesting for routine analysis because the analysis time can be reduced from 20 h to about 2 h. However, this will require improvements in sample preparation so that natural fibers can be fully digested or, at least broken down into tiny particles. Otherwise, there is a risk that MPs could be trapped by fragments of natural fibers, and eventually, not recognized as individual particles. Once this requirement is met, it would be possible to compare the automatic particle location software and the mapping approach to assess the pros and cons of both approaches.

The SEM–EDX analysis corroborates the results obtained with Raman for the cow’s milk and water samples spiked with MPs regarding their physical integrity. However, the elemental composition analysis by EDX was not sufficiently discriminative to confirm that the microparticle analyzed was a MP or an organic residue from the digestion of cow's milk. Therefore, EDX was undoubtedly a powerful tool to support Raman, but it cannot be used as a confirmatory technique for the correct identification of organic compounds such as MPs.

The Raman method used was performant in detecting and identifying all types of MPs ≥ 5 µm size. The exogenous MPs detected by Raman in samples of cow's milk had a spectral profile very similar to the MP standards but was significantly less intense. This difference may be related to the fact that exogenous MPs are exposed to environmental agents (e.g. sunlight, temperature, humidity, etc.) and various processing steps used to convert farm’s milk into milk-based products. Despite this reduction in intensity of the peaks of the spectrum, the Raman method developed was sufficiently sensitive and specific to correctly detect and identify MPs with dimensions ≥ 5 µm.

The lowest number of MPs was generally found in liquid milk samples except one raw milk collected at farm level. PE, the main polymer present in the milking machine, were the likely contributor to MPs contamination of the raw milk. The origin of other main MPs like PP, PES and PTFE in raw milk can be multiple, e.g. ubiquitous presence in the farm environment and along milking process including storage containers. Depending on the type of packaging, PP particles could likely come from bottle (processed liquid milk—brand C) and PE from multilayer laminated paper (processed liquid milk samples—brands A and B). Three other types of MP (PU, PSU and PVA) were detected in liquid milk samples (brand B). The presence of PSU is a potential indication of plastic debris originating from the abrasion of membrane filters used during milk processing^35^. PVA can come from the packaging, since PVA is usually used as oxygen and odor barrier polymer in multilayer laminated paper. The custom-made software detected the presence of PSU and PVA, although they were not included in the polymer database. Initially, these polymers were incorrectly classified as another type of polymer. However, the incorrect assignment was detected immediately, since the spectrum associated with each particle appears in the analytical report. Subsequently, the identification of these polymers was done using the KnowItAll software and the Raman library from Bio-Rad.

Powdered milk samples showed the highest number of MPs (PP, PE, PES and PTFE). These types of MPs are the most ubiquitous in environment and their presence in milk samples may be due to environmental contamination, milking process (i.e. series of macro, micro and ultrafiltration using polymeric membranes and additional drying steps for powder) and packaging conditioning from farms to dairy processing facility. A large number of natural microfibers of various sizes were identified as cellulose (Fig. S3). On the other hand, the presence of large synthetic microfibers was not observed in samples of liquid cow’s milk and powdered milk. Two reasons may explain the absence of large synthetic microfibers commonly found in food matrices: firstly the contamination prevention protocol used in this study and secondly the microfiltration of the milk used at the farm and then at the factory (> 100 µm) to remove foreign bodies. In addition, the milk can be subjected to microfiltration (pore sizes ranging from 0.1 to 10 µm) and ultrafiltration (pore sizes ranging from 3 to 50 nm) at the factory. Microfiltration and ultrafiltration are used to extend the milk shelf life by removing bacteria and spores. Ultrafiltration is often used in the milk process to increase the protein concentration while reducing the concentration of sugars and removing lactose. However, it is important to highlight that the filter can be made either of ceramic, or a mixture of polymers (cellulose, cotton, viscose and polyester) with specific physical characteristics. These polymeric filters are traditionally white, but colored membrane filters are also available in some countries to help identify white spots of mastitic milk. Therefore, the risk of the white and colored fibers of the polymers being released by the filter cannot be ruled out. Thus, the presence of small fragments of microfibers cannot be excluded even if large microfibers were not detected.

The Raman analysis of dark and grey MPs may not be feasible because it generally shows low signal intensity and no specific polymer peaks. This phenomenon is due to the absorption of laser light by carbon black. The vast majority of food contact material is transparent, translucent or slightly colored. However, the risk of environmental contamination with colored microfibers cannot be eliminated. The colored microfibers can be very challenging to be analyzed by Raman technology because the Raman spectrum peaks from pigments are generally more intense than those from polymers. As a result, microfibers with intense colors may need to be analyzed by micro-infrared spectroscopy (but generally for size of MPs > 20 µm) so that the synthetic polymer can be identified. For such cases, the color and type of synthetic polymer may be the key to a successful root cause analysis. In contrast to the previous works^35,36^ where an average of 6 MPs/L ^35^ and 40 MPs/L ^36^ of milk samples (mainly sulfone thermoplastics, PP and PE particles with size > 10 µm) were identified by µFTIR and µRaman, our study detected a higher average number and types of MPs. The most abundant MPs determined were PP, PE, PES and PTFE with a size < 7 µm (i.e. surface area ≤ 50 µm^2^). The higher number of MPs detected in this study may be associated with the fact that a point-by-point Raman approach was able to detect smaller particles in milk products than those detected by the visual detection approach of MPs > 10 µm size adopted in previous studies^35,36^.

Assuming that MPs are considered as migrating polymers with a rough estimation that 100% MPs are spherical particles of 10 µm diameter with a density of ca 1 g/cm^3^, a number of 1000 MPs found per serving size (i.e. 100 mL) in the milk samples corresponds to a concentration of MPs of ca 5 µg per kg of product. Even if a more accurate calculation might be applied for MP sizing using the approach proposed by Simon et al.^60^, the rough MPs concentration estimated in this study is significantly lower than the overall limit of substance migration (60 mg/kg food) and the specific migration of oligomeric fraction (less than 1000 Da) of polymers (50 µg per kg food) specified in the EU N° 10/2011 related to plastic migration^61^. However, the size and type of MPs found in foodstuffs have also to be assessed from a toxicological point of view which is the subject of numerous project proposal for funding by different authorities around the world.

Thus, it is urgent to standardize appropriate isolation and identification methods of MPs in food that follow guidelines using reliable protocols with strict quality assurance including control measures of method blanks and MPs recovery in order to provide sensitive, accurate and comparable results^62–64^. Due to the different nature and size of MPs in a great range of food matrices, many efficient extraction methods using density separation and chemical or/and enzymatic digestions before filtration steps are available but need to be improved for better MPs recovery without degrading MPs using quicker sample treatments^65^. Each analytical method has its strengths and weaknesses for MPs characterization at micrometer level even if µRaman or µFTIR spectroscopy present more advantages in terms of sensitivity and specificity than SEM, flow cytometry, staining techniques or destructive method like Py-GC-MS^45^.

## Conclusion

A new validated methodology for isolation and detection of MPs in milk samples was successfully used for the first time to detect and identify micro-sized MP (≥ 5 µm) in raw and processed cow’s milk.

This study has shown a slight trend of increase of MPs with the degree of milk processing and packaging conditions. Therefore, this method could be a good analytical tool for route cause analysis of MPs contamination along the dairy chain and therefore to mitigate the number and types of MPs in cow’s milk products. Additionally, it could be further used by multinational corporations in a worldwide survey of dairy products and related ingredients to support the international authorities in performing toxicological risk assessments of human diets. However, the method must first be standardized to be further accepted worldwide by international authorities. Therefore, in view of presenting the method for evaluation as an official method, it will be recommended to extend the validation to a broader list of polymers (e.g. PET /PES, PU, PTFE, PVC, etc.).

Finally, with complementary work to optimize µRaman analysis time using a rapid particle counting approach, this could lead to an acquisition time of only 2–3 h versus ca 20 h in the current setup. In such case, this could contribute to a mandatory standardization of MPs determination in other foodstuffs including raw materials in order to ensure trust between authorities, food industry and consumers.

## Method and materials

### Cow’s milk sampling

Some of the most commonly sold milk products in Switzerland were purchased in stores from 3 main supermarket chains. Three whole liquid milk, one skimmed liquid milk and two skimmed milk powders were collected and stored in the laboratory at 6 °C. Raw milk was first collected at a farm in France (Jura country), where the samples were collected from the milking machine (Milk Master, DeLaval) directly into a glass container (250-mL, particle certified (i.e. with less than 5 particles (< 0.5 µm) per mL), ThermoFisher Scientific) on two different days, then transported in a cold container to Switzerland and finally stored at 6 °C in our laboratory before analysis. The samples used for this study are listed in the Table S2.

### Sample preparation

#### Milk sample digestion

Powdered milk samples were initially reconstituted with ultrapure water in a glass flask (1000-mL; particle certified, Thermofisher Scientific) by diluting 25 g test portion in 175 g ultrapure water (Lichrosolv, Merck). Glass flasks covered with a glass lid were shaken for at least 15 min at 40 °C into a shaking water bath (GFL-1083, Milian). Processed liquid and raw milk samples were used as is.

25 mL liquid or reconstituted milk sample and 20 mL ultrapure water (Lichrosolv, Merck) were added into a glass flask (1000-mL; particle certified, ThermoFisher Scientific); 2 mL of multi-enzymatic detergent (Prozyme, Borer Chemie AG) was added and mixed for 2 min at 40 °C in the container; 10 mL of calcium chelating agent sodium ethylene diamine tetra acetate (EDTA-Na 0.5 M, pH 8, Invitrogen, ThermoFisher Scientific) was added and mixed for 3 min at 40 °C in the container; 2 mL of alkaline solution tetramethyl ammonium hydroxide (TMAH 25% v/v Sigma-Aldrich) was added into the glass container that was finally immediately put in a microwave (CD575MWPG, Panasonic NN) at a power of 1000 watts for a maximum of 1 min to ensure a final temperature below 80 °C. The hot digested milk sample was then immediately submitted to filtration process.

#### Milk sample filtration

Just after microwave heating, hot digested milk sample was directly poured into a glass funnel (100-mL, Sterlitech) mounted onto a filtration unit (glass holder with13 mm frit, stainless steel vacuum manifold, Rocker 400 Vacuum Pump, 220 V/50 Hz, Sterlitech). Contents of the funnel pass through a Si filter (Silicon filter, 10 × 10 mm size, 500 μm thickness, 5 μm porosity, SmartMembrane) under a vacuum for a maximum of 5 min. Custom-made filter holder (stainless steel, 10 × 10 mm, 25 mm), rubber hole seals (ethylene propylene diene monomer (EPDM), 25 mm diameter, 2 mm thickness, 4 and 8 mm diameter holes), seal holder and cover (stainless steel, 25 mm, 8 mm diameter hole) were used for a restricted filtration area of ca 14 mm^2^ (i.e. ca 4 mm diameter area) on silicon filter (Fig. S1). Retained digested material was successively flushed with first 5 mL water then 5 mL diluted nitric acid 5% v/v and finally 10 mL water before being stored in a closed glass container before µRaman analysis.

### Microplastics analysis

#### Mitigating laboratory contamination

A major challenge in the measurement of MPs is to avoid contamination of the test sample when handled in the broader laboratory environment, that includes, for example, particles from the ambient air, clothing, utensils, chemicals, and so forth. Meticulous study of the different possible steps that may introduce “foreign” MPs is a prerequisite to ensure reliable measurement. Therefore, emphasis on lowering contamination of the method blanks (i.e. blank following all the method process but omitting the sample matrix) besides the usual method validation parameters is pivotal. Cotton lab coats and powder-free disposable gloves (Microflex Neotouch, Ansell) were systematically used. Class 2 biosafety cabinet (CytoFast Elite, Faster) with certified ‘low pressure-drop’ H14 HEPA/ ULPA filters, filtration unit, dedicated glassware and spectroscopy equipment (µRaman and optical microscopy) were installed in clean laboratory rooms (ISO class 5) with positive air pressure.

Control and verification of clean laboratory environment for sample preparation was performed before this study measuring MPs retained on some silicon filters put on laboratory benches and in biocabinet. Grade air purifier (APS-500, Kynio) and particle counter (PC 220, Trotec) were also used during the study in the laboratory for mitigating and daily monitoring particles respectively. In addition, method blanks (i.e. blanks following all the method process but omitting the sample matrix) were systematically analyzed for each series of analysis.

Concentrated alkaline solution (TMAH) is pre-filtered through chemically resistant 5 μm silver filter (Sterlitech) whereas all other reagents used for milk preparation (i.e. multi-enzymatic detergent solution, EDTA-Na, ethanol 70% v/v and ultrapure water) are pre-filtered through 0.65 μm polyvinylidene difluoride (PVDF) filter (Merck) using stainless steel filter holders (Merck). Specific 1-L or 250-mL Particle Free (PF) glass bottles (Thermo) are used for digestion procedure.

All the glass vessels (funnel, beakers, glass bottles), restrictor seals and filter holders were pre-cleaned thoroughly with ethanol 70% v/v and rinsed 3 times with ultrapure water (Merck).

The Si filter was pre-cleaned in a glass container with a pre-filtered aqueous solution of ready-to-use multi-enzymatic detergent (Prozyme, Borer Chemie AG) 10% v/v and finally rinsed with pre-filtered ultrapure water (Merck) in another glass flask. All the reagents used in filtration step (i.e. water, HNO_3_ (Merck) were pre-filtered on 0.65 μm PVDF filter (Merck)).

#### Digestion efficiency and method recovery

The 2 quality criteria used for both digestion and filtration steps were the digestion efficiency and recovery rates of spiked polymers in water and real sample.

Digestion efficiency was verified by counting remaining digested particles and the determination of total related surface covered in the filter by the digested matrix. The particle counting and determination of surface covered was performed using a digital optical microscope VHX-6000 (Keyence). Preliminary tests on procedural blanks have shown that less than 300 retained particles per mm^2^ of filtration area (corresponding to a maximum of 5% surface covered) was obtained resulting in a low contamination of MPs coming from reagents, filtration unit, glassware and laboratory environment.

A maximum limit of 600 retained particles per mm^2^ of filtration area (corresponding to a maximum of 30% surface covered) was set for a digest sample in order to ensure that the sample was digested efficiently. This last quality criterion must be fulfilled to ensure that the filter was not overloaded with too many organic residues, which could potentially hide MPs during Raman analysis or even clog the filter.

Raman method was used to determine the MPs recovery rate that was calculated by spiking one ultrapure water without alkaline digestion step before direct filtration and one cow’s milk sample (brand B, whole milk, 3.5% fat) following the whole procedure of digestion and filtration. In addition, 4 samples of cow's milk (brand B, whole milk 2, 3.5% fat) spiked with the standard solutions of MPs were prepared to assess the extrapolation factor applied to estimate the number of MPs retained on the entire filter. These samples followed the entire digestion and filtration procedure. Different MP standards in solution (PMMA (90515-5ML-F), PS (89756-5ML-F) and PA (GF71024565), Sigma-Aldrich) and powder (PE (FIPOLDER FHP0204) and PP (PROPOLDER FPP4010) from TWO H Chem Ltd) were internally prepared and calibrated by spectral flow cytometry (FC, Cytek – Nestlé Research, Lausanne) in water containing sodium dodecyl sulfate detergent (SDS, Invitrogen) at 5% m/v. These microplastics standards were selected because of commercial availability and some of them are frequently used in packaging and/or food contact materials (e.g. PP, PE, and PS). Polymer standard solutions used for recovery rates are listed in Table S3.

#### SEM imaging—EDX analysis for polymer integrity

Scanning electron microscope (Quattro S, Thermofischer) equipped with an Energy Dispersive X-Ray Spectroscopy detector (Xmax 50 mm^2^, Oxford Instruments) was used to check the quality of digestion and the physico-chemical integrity of the MPs on one spiked ultrapure water and one spiked cow milk sample (brand C, skimmed milk, 0.1% fat). SEM micrographs of the Si filter surface combined with EDX analysis of the encountered particles were acquired for 2 spiked samples in order to obtain simultaneous particle imaging and elemental chemical composition.

Prior to analysis, Si filters were coated with either 5 nm of gold (for imaging purposes) or with 5 nm of carbon (for combined imaging and chemical composition).

Imaging of single particles was performed at an accelerating voltage of 5 kV in secondary electron and backscattered electron modes.

EDX spectra were acquired on single particles at 20 kV to confirm the organic or mineral nature of particles and subsequently at 5 kV to improve C, O and N detection. A minimum of 300,000 counts was acquired per measurement.

#### Validation of Raman methodology

Spiking experiment of a processed cow’s milk sample (brand C, skimmed milk, 0.1% fat) using Raman method was performed using different polymer standard solutions (PMMA, PS, PA, PE and PP) internally prepared and calibrated by flow cytometry (FC) in water containing sodium dodecyl sulfate detergent (SDS, Invitrogen) at 5% m/v with the polymer concentrations displayed in Table S3. Optical image of a part of Si filter and Raman analysis of some retained MPs were performed to check the possible changes in fingerprint spectra due to a potential degradation of MPs after alkaline digestion step.

#### Raman analysis

A confocal μRaman Labram HR Evolution (Horiba, SAS France, Villeneuve d’Ascq, France) equipped with an EMCCD detector, 50 × magnification long working distance objective (Olympus, NA = 0.5), dark field system and 532 nm solid-state laser (power 50 mW), acquisition time of 0.1 s in a spectral range of 550 cm^−1^ to 2000 cm^−1^ (resolution of 4 cm^−1^) and 10 mW of laser power was focused into the sample to acquire the Raman spectrum. This instrument setting generated a laser beam of ca 1 µm.

In this approach, 2 areas representing ca 50% of total filter surface (i.e. 7 mm^2^) were consecutively analyzed (Fig. S2). The instrument takes 2 measurement times of 10 h to perform point-by-point mapping with step of 5 μm with a final acquisition of about 250,000 Raman spectra.

#### Raman data processing

After Raman spectra acquisition using LabSpec 6.5 software, a custom-made software was developed for identification and characterization of microparticles. The training data set was constructed using Raman spectra collected from exogenous MPs found in food samples and commercial MPs spike-in milk products. Such diverse set of MPs sources allowed a better representation of the possible range of signals for each given polymer. Furthermore, a wide range of signal quality was selected to better represent the noisier signals that can be found in these samples. The identity of all polymer spectra included in the training set was confirmed manually using the Bio-Rad database. The training set was composed using 12,549 spectra of 9 different types of plastic (PA, PE, PES, polylactic acid (PLA), PMMA, PP, PS, PU and PTFE) with addition of stearates and other non-microplastic particles (NMP) such as organic residues from matrix decomposition and spectrum of silicon filter. Because stearates can be found in lab gloves and display a very similar signal to PE^66^, they were added as a separate class. In this way, it is easier to identify potential miss-classification between the two classes. To better fit our applications, it would be more appropriate to merge it into the NMP class. Table 3 shows the number of spectra per class in the training set.

**Table 3:** Number of spectra for each type of class used in the training set.

Class	NMP	PA	PE	PES	PLA	PMMA	PP	PS	PTFE	PU	Stearate
Number	4146	635	807	726	1638	391	758	567	903	994	984

The following steps describe the data pre-processing applied to build the database and to analyze the samples. The spectra database contained few spectra with slightly different spectral range. However, this small variation in the spectral range is not compatible with most machine learning algorithm, including random forest. Therefore, the spectra range and measurement points were refitted (559 cm^−1^ to 1990 cm^−1^, every 3 cm^−1^) using R version (4.0.2) stats splinefun function to allow consistent measurement points of the spectra. Baseline correction was performed using the R package “baseline” version (1.3-1) to remove the background noise from the spectrum^67^. A set of “universal” parameters was chosen to best fit the observed cases. To reduce heavy fluctuation of the signal, the spectra were smoothed using another spline function included in the R package “hyperSpec” version (0.99-20171005)^68^. Parameters were chosen so that all data points were used with a reduced smoothing parameter to avoid any degradation of the signal. Finally, the spectra values were scaled over their respective standard deviation.

The Random Forest algorithm from Ranger version (0.12.1)^69^ is trained not only to distinguish the plastic types as proposed by Vinay Kumar et al.^70^ but also to identify other types of materials. The Random Forest machine learning algorithm was trained with the processed data 1500 estimators (trees) and will classify each spectrum in one of the MP classes or in the non-MP class^71^. The prediction error of Random Forest was estimated using out-of-bag (OOB) error. After being automatically classified, the spectrum with low confidence score are controlled by the user to avoid miss classification. Tables S6 and S7 show the confusion matrix and the percentage of samples incorrectly classified in the training dataset, respectively.

Using the physical position of each measurement, the neighboring signals of the same class of polymer were grouped into particles. Using the R package “lattice” version (0.20-41)^72^, a graph represented the physical connections between spectra. The edges of the graph were then kept if the maximum distance between two spectra of a same polymer class was two. Effectively, this allowed clustering of polymer signals based on their relative physical location, while jumping over “missed” signals. Missed signal could be caused by a short desynchronization between the computer clock and the speed of acquisition of the spectrum.

## Supplementary Information

Supplementary Information 1.

## Data Availability

The script and datasets generated or analyzed during this study are available in the GitHub and Zenodo repositories. https://doi.org/10.5281/zenodo.5607596. https://github.com/BenoitCarreres/Nestle_microplasticAnalyzer.: Tool developed by Société des Produits Nestlé S.A. (Nestlé Research) to process Raman spectra (Horiba) data and identify microplastic particles. (github.com).
