# Supplementary material for: Detection and characterization of small-sized microplastics (≥ 5 µm) in milk products
Source: Sci Rep. 2021 Dec 15;11:24046. doi: 10.1038/s41598-021-03458-7 (PMC8674347; doi:10.1038/s41598-021-03458-7)
Supplement: Supplementary file 1 — Supplementary Information 1. [file 41598_2021_3458_MOESM1_ESM.docx]

**Supplementary Information**

**Detection and characterization of small-sized microplastics (≥ 5 µm) in milk products**

**Paulo A. Da Costa Filho, Daniel Andrey, Bjorn Eriksen, Rafael Peixoto, Benoit M. Carreres, Mark E. Ambühl, Josep Busom Descarrega, Stephane Dubascoux, Pascal Zbinden, Alexandre Panchaud & Eric Poitevin**

Société des Produits Nestlé S.A. Nestlé Research, Route du Jorat 57, Lausanne, Switzerland.

Correspondence and requests for materials should be addressed to P.A.D.C.F (PauloAugusto.DaCostaFilho@rdls.nestle.com).

**Table S1. Raman band assignment of PMMA, PS, PP, PE and PA. (1-4)**

| PMMA | | PS | | PP | | PE | | PA | | |
| --- | --- | --- | --- | --- | --- | --- | --- | --- | --- | --- |
| Raman shift/cm^-1^ | Mode | Raman shift/cm^-1^ | Mode | Raman shift/cm^-1^ | Mode | Raman shift/cm^-1^ | Mode | Raman shift/cm^-1^ | Mode |  |
| 814 | ν (C=O) | 621 | Φ_d_ | 808 | r (CH_2_)  ν (C-C) | 1063 | ν_as_ (C-C) | 1636 | ν_s_ (C=O) |  |
| 1730 | ν_as_ (C-O-C) | 1001 | Φ_b_ | 841 | r (CH_2_) | 1080 | ν (C-C) | 1466 | ν (C-N) & δ (NH) |  |
|  |  | 1031 |  | 972 | r (CH_3_)  ν (C-C) | 1130 | ν_s_ (C-C) | 1442 | δ (CH_2_) |  |
|  |  | 1155 | ν (C-C) | 998 | r (CH_3_) | 1298 | ν_t_ (C-C) | 1307 | ν_t_ (CH_2_) |  |
|  |  | 1450 | δ (CH_2_) | 1151 | ν (C-C)  δ (CH) | 1418 | δ (CH_2_)  ω (CH_2_) | 1280 | ν (C-N) & δ (NH) |  |
|  |  | 1583 | ν (C=C) | 1168 | ν (C-C)  r (CH_3_)  ω (C-C) | 1440 | δ (CH_2_) | 1126 | ν (C-C) |  |
|  |  | 1602 | Φ_s_ | 1220 | ν_t_ (C-C)  ω (C-H)  ν (C-C) | 1460 | δ (CH_2_) | 1078 | ν (C-C) |  |
|  |  |  |  | 1435 | δ (CH_2_) |  |  | 1062 | ν (C-C) |  |
|  |  |  |  | 1458 | δ (CH_2_) |  |  | 931 | ν (C-CO) |  |

r rocking; Φ_d_ ring deformation_,_ Φ_b_ ring breath; ν stretching; Φ_s_ ring stretching, ν_as_ anti-symmetric stretching; ν_s_ symmetric stretching; ν_t_ twisting; δ bending, ω wagging.

**Table S2. List of milk samples used for this study**

| **Sample Description** | **Type** | **Amount** | **Packaging** | **Collection Date** |
| --- | --- | --- | --- | --- |
| Raw milk (milking machine) - 1^st^ collection | Liquid | 200 mL | PF glass bottle | 13.03.2019 |
| Raw milk (milking machine) - 2^nd^ collection | Liquid | 200 mL | PF glass bottle | 25.04.2019 |
| Brand A, whole milk (3.5% fat) | Liquid | 250 mL | Brick | 25.02.2019 |
| Brand B, whole milk 1 (3.9% fat) | Liquid | 250 mL | Brick | 25.02.2019 |
| Brand C, whole milk (3.5% fat) | Liquid | 500 mL | PP Bottle | 25.02.2019 |
| Brand A, skimmed milk | Powder | 350 g | PE Bag | 04.05.2020 |
| Brand B, skimmed milk | Powder | 350 g | PE Bag | 04.05.2020 |
| Brand B, whole milk 2 (3.5% fat) (spiking experiment for recovery and extrapolation factor - Raman analysis) | Liquid | 500 mL | Carton brick | 28.05.2020 |
| Brand C, skimmed milk (0.1% fat) (spiking experiment for polymer integrity - SEM-EDX and Raman analysis) | Liquid | 1000 mL | Carton brick | 08.09.2020 |

**Table S3. Concentrations of polymer standard solutions and related physical properties**

| **Polymer Type** | **Shape** | **Diameter (µm)** | **Spiked Mother Solutions**  **Concentration (MPs/µL)** |
| --- | --- | --- | --- |
| PMMA | Spherical | 8 | 1045 |
| PS | Spherical | 6 | 652 |
| PP | Fragment | 5-20 | 157 |
| PE | Fragment | 5-20 | 74 |
| PA | Fragment | 5-50 | 348 |


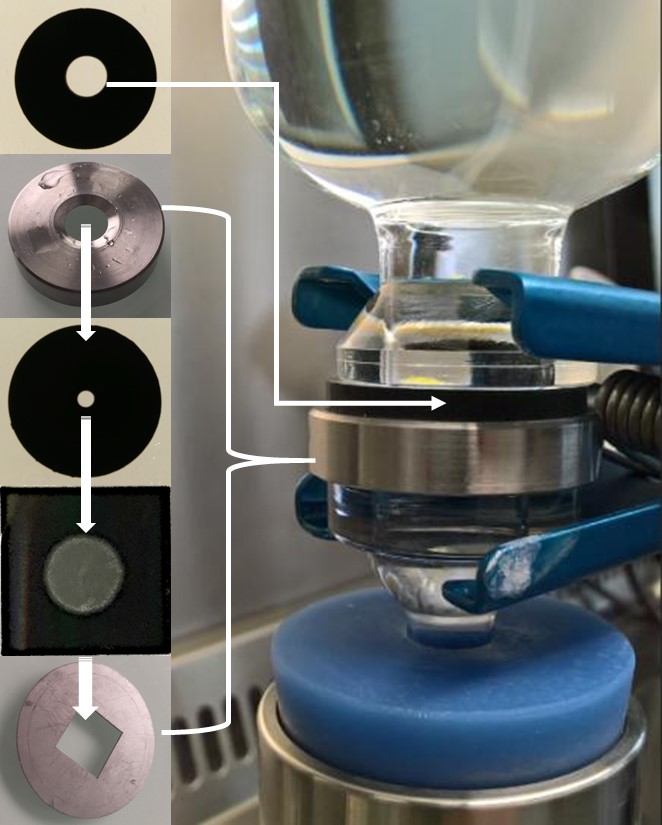


**Figure S1. Custom-made filter mounting system for MPs isolation.**


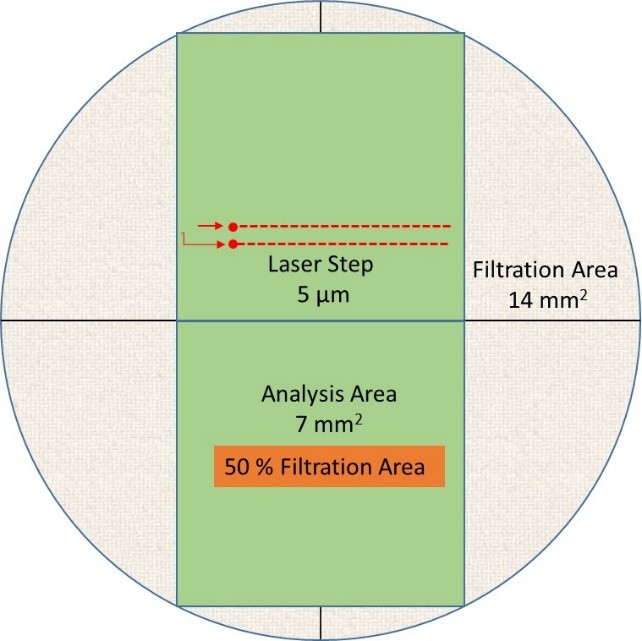


**Figure S2. Filtration area (14 mm^2^) used for µRaman point by point approach method with a step of 5 µm**


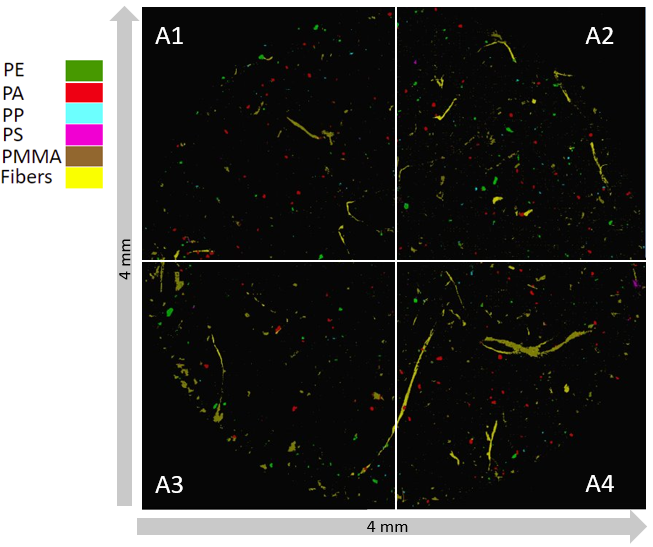


**Figure S3. µRaman analysis of entire filtered surface for a cow’s milk (brand B, whole milk 2, 3.5% fat)**

Table S4. Number of particles of each type of microplastics founds in 25% and 100% of the filtered area.

|  | **Sub-areas** | **PA** | **PE** | **PMMA** | **PP** | **PS** |
| --- | --- | --- | --- | --- | --- | --- |
| **Replicate 1** | Sub-area 1 | 37 | 63 | 53 | 73 | 34 |
|  | Sub-area 2 | 32 | 81 | 36 | 68 | 35 |
|  | Sub-area 3 | 28 | 83 | 44 | 69 | 21 |
|  | Sub-area 4 | 45 | 49 | 41 | 46 | 40 |
|  | **Total** | **142** | **276** | **174** | **256** | **130** |
| **Replicate 2** | Sub-area 1 | 35 | 63 | 29 | 60 | 29 |
|  | Sub-area 2* | 46 | 68 | 48 | 76 | 38 |
|  | Sub-area 3 | 45 | 60 | 42 | 62 | 37 |
|  | Sub-area 4 | 43 | 66 | 44 | 95 | 25 |
|  | **Total** | **169** | **257** | **163** | **293** | **129** |
| **Replicate 3** | Sub-area 1 | 38 | 79 | 29 | 80 | 29 |
|  | Sub-area 2 | 40 | 75 | 36 | 78 | 23 |
|  | Sub-area 3 | 36 | 76 | 46 | 65 | 25 |
|  | Sub-area 4 | 32 | 51 | 33 | 56 | 20 |
|  | **Total** | **146** | **281** | **144** | **279** | **97** |
| **Replicate 4** | Sub-area 1 | 37 | 88 | 48 | 67 | 41 |
|  | Sub-area 2 | 40 | 99 | 31 | 83 | 35 |
|  | Sub-area 3 | 33 | 86 | 38 | 66 | 21 |
|  | Sub-area 4 | 42 | 90 | 38 | 80 | 27 |
|  | **Total** | **152** | **363** | **155** | **296** | **124** |

(*) This file is corrupted, and the values presented in the manuscript are the extrapolation from the partial area analyzed.

Table S5. Ratio between the number of particles of a given polymer found in 100% and in 50% of the filtered area.

|  | **Sub-areas combinations** | **PA** | **PE** | **PMMA** | **PP** | **PS** |
| --- | --- | --- | --- | --- | --- | --- |
| **Replicate 1** | A1 & A2 | 2.06 | 1.92 | 1.96 | 1.82 | 1.88 |
|  | A1 & A3 | 2.18 | 1.89 | 1.79 | 1.80 | 2.36 |
|  | A1 & A4 | 1.73 | 2.46 | 1.85 | 2.15 | 1.76 |
|  | A2 & A3 | 2.37 | 1.68 | 2.18 | 1.87 | 2.32 |
|  | A2 & A4 | 1.84 | 2.12 | 2.26 | 2.25 | 1.73 |
|  | A3 & A4 | 1.95 | 2.09 | 2.05 | 2.23 | 2.13 |
|  | **Mean ± STD** | **2.02 ± 0.23** | **2.03 ± 0.27** | **2.01 ± 0.18** | **2.02 ± 0.21** | **2.03 ± 0.28** |
| **Replicate 2** | A1 & A2 | 2.09 | 1.96 | 2.12 | 2.15 | 1.93 |
|  | A1 & A3 | 2.11 | 2.09 | 2.30 | 2.40 | 1.95 |
|  | A1 & A4 | 2.17 | 1.99 | 2.23 | 1.89 | 2.39 |
|  | A2 & A3 | 1.86 | 2.01 | 1.81 | 2.12 | 1.72 |
|  | A2 & A4 | 1.90 | 1.92 | 1.77 | 1.71 | 2.05 |
|  | A3 & A4 | 1.92 | 2.04 | 1.90 | 1.87 | 2.08 |
|  | **Mean ± STD** | **2.01 ± 0.13** | **2.00 ± 0.06** | **2.02 ± 0.22** | **2.02 ± 0.25** | **2.02 ± 0.22** |
| **Replicate 3** | A1 & A2 | 1.87 | 1.82 | 2.22 | 1.77 | 1.87 |
|  | A1 & A3 | 1.97 | 1.81 | 1.92 | 1.92 | 1.80 |
|  | A1 & A4 | 2.09 | 2.16 | 2.32 | 2.05 | 1.98 |
|  | A2 & A3 | 1.92 | 1.86 | 1.76 | 1.95 | 2.02 |
|  | A2 & A4 | 2.03 | 2.23 | 2.09 | 2.08 | 2.26 |
|  | A3 & A4 | 2.15 | 2.22 | 1.82 | 2.31 | 2.16 |
|  | **Mean ± STD** | **2.00 ± 0.10** | **2.02 ± 0.20** | **2.02 ± 0.22** | **2.01 ± 0.18** | **2.01 ± 0.17** |

The confusion matrix of the training of the algorithm is shown in Table S6. The model correctly identified and categorized microplastics with a high level of confidence. The Random Forest model showed a prediction error of 0.0091 using the out of bag (OOB) approach. In addition, the model was able to discriminate similar spectra, such as stearates and PE, without much confusion during the OOB forecasting process.

Table S6. Confusion matrix of the Random Forest training.

|  |  | Predicted | | | | | | | | | | |
| --- | --- | --- | --- | --- | --- | --- | --- | --- | --- | --- | --- | --- |
|  | **Class** | **NMP** | **PA** | **PE** | **PES** | **PLA** | **PMMA** | **PP** | **PS** | **PTFE** | **PU** | **Stearate** |
| T  R  U  E | **NMP** | 4100 | 3 | 9 | 3 | 0 | 1 | 11 | 2 | 11 | 5 | 1 |
|  | **PA** | 4 | 631 | 0 | 0 | 0 | 0 | 0 | 0 | 0 | 0 | 0 |
|  | **PE** | 7 | 0 | 797 | 0 | 0 | 0 | 0 | 0 | 0 | 0 | 3 |
|  | **PES** | 1 | 0 | 0 | 725 | 0 | 0 | 0 | 0 | 0 | 0 | 0 |
|  | **PLA** | 1 | 0 | 0 | 0 | 1637 | 0 | 0 | 0 | 0 | 0 | 0 |
|  | **PMMA** | 1 | 0 | 0 | 0 | 0 | 390 | 0 | 0 | 0 | 0 | 0 |
|  | **PP** | 21 | 0 | 0 | 0 | 0 | 0 | 737 | 0 | 0 | 0 | 0 |
|  | **PS** | 0 | 0 | 0 | 0 | 0 | 0 | 0 | 567 | 0 | 0 | 0 |
|  | **PTFE** | 25 | 0 | 0 | 0 | 0 | 0 | 0 | 0 | 878 | 0 | 0 |
|  | **PU** | 10 | 0 | 0 | 0 | 0 | 0 | 0 | 0 | 0 | 984 | 0 |
|  | **Stearate** | 2 | 1 | 20 | 0 | 0 | 0 | 0 | 0 | 0 | 0 | 961 |

Table S7. Sensitivity, specificity, percentage of false positive and false negative results for each class of material present in the database. (5)

|  | NMP | PA | PE | PES | PLA | PMMA | PP | PS | PTFE | PU | Stearate |
| --- | --- | --- | --- | --- | --- | --- | --- | --- | --- | --- | --- |
| Sensitivity | 98.46 | 99.63 | 98.79 | 99.88 | 100 | 99.88 | 97.51 | 100 | 97.68 | 99.30 | 97.76 |
| False positive (%) | 1.54 | 0.37 | 1.21 | 0.12 | 0 | 0.12 | 2.49 | 0 | 2.32 | 0.70 | 2.24 |
| Specificity | 99.34 | 99.97 | 99.74 | 99.98 | 100 | 99.99 | 99.84 | 99.99 | 99.91 | 99.96 | 99.98 |
| False negative (%) | 0.66 | 0.03 | 0.26 | 0.02 | 0 | 0.01 | 0.16 | 0.01 | 0.09 | 0.04 | 0.02 |

**References**

1. Miller, J. V. & Bartick, G. E., Forensic analysis of single fibers by Raman spectroscopy. *Applied Spectroscopy,* 55, 12, 1729-1732 (2001).

2. Kida, T., Hiejima, Y. & Nitta, K-h., Raman spectroscopy study of high-density polyethylene during tensile deformation. *Int. J. Exp. Spectroscopic Tech*. 1-6, (2016).

3. Nielsen, A. S., Batchelder, D. N. & Pyrz, R., Estimation of crystallinity of isotactic polypropylene using Raman spectrsocopy. *Polymer*. 43, 2671-2676, (2002).

4. Hu, C., Chen, X., Chen, J. Zhang, W. & Zhang, M. Q., Observation of mutual diffusion of macromolecules in PS/PMMA binary films by confocal Raman microscopy. *Soft Matter*, 8,17, 4780-4787 (2012).

5. Trullols, E., Ruisánchez, I., Xavier Rius, F., Validation of qualitative analytical methods. *Trends Anal. Chem.* 23, 2, 137-145 (2004)
